# A new tool for the paediatric HIV research: general data from the Cohort of the Spanish Paediatric HIV Network (CoRISpe)

**DOI:** 10.1186/1471-2334-13-2

**Published:** 2013-01-02

**Authors:** Ma Isabel de Jose, Santiago Jiménez de Ory, Maria Espiau, Claudia Fortuny, Ma Luisa Navarro, Pere Soler-Palacín, Ma Angeles Muñoz-Fernandez

**Affiliations:** 1 Servicio Infecciosas Infantil, Hospital Universitario “La Paz”, Paseo de la Castellana 26, Madrid, 128046, Spain; 2 Laboratorio de Inmuno-Biología Molecular. Spanish HIV HGM BioBank. Hospital General Universitario “Gregorio Marañón” and Instituto de Investigación Sanitaria Gregorio Marañón, C/Dr. Esquerdo 46, Madrid, 28007, Spain; 3 Unitat de Patologia Infecciosa i Immunodeficiències de Pediatria, Hospital Universitari Vall d` Hebron, Universitat Autònoma de Barcelona, Barcelona, Spain; 4 Servicio Infecciosas Infantil, Hospital Sant Joan de Dèu, Esplugues de Llobregat, Barcelona, Spain; 5 Sección de Enfermedades Infecciosas. Servicio de Pediatría. Hospital General Universitario “Gregorio Marañón”, Madrid, Spain

**Keywords:** HIV paediatric cohort, Paediatric HIV infection, Spanish HIV HGM biobank

## Abstract

There are approximately from 1,100 to 1,200 HIV-infected children in a follow-up in Spain. In 2008 an open, multicentral, retrospective and prospective Cohort of the Spanish Paediatric HIV Network (CoRISpe) was founded. The CoRISpe is divided into the node 1 and node 2 representing geographically almost the whole territory of Spain. Since 2008 seventy-five hospitals have been participating in the CoRISpe. All the retrospective data of the HIV-infected children have been kept in the CoRISpe since 1995 and prospective data since 2008. In this article we are going to present the notion of CoRISpe, its role, the structure, how the CoRISpe works and the process how a child is transferred from Paediatric to Adults Units.

The main objective of the CoRISpe is to contribute to furthering scientific knowledge on paediatric HIV infection by providing demographic, sociopsychological, clinical and laboratory data from HIV-infected paediatric patients. Its aim is to enable high-quality research studies on HIV-infected children.

## Background

Current treatment to prevent mother-to-child transmission (MTCT) of HIV is very different from that a few years ago [1]. In the first years of HIV infection there were not treatments for HIV-infected children [2,3]. In the years from 1985 to 1996 children were treated with monotherapy or combination therapy [4]. Since then, highly active antiretroviral therapy (HAART) has been used [5-9]. Spain has had free general access to HAART since 1996. By the end of 2011 there have been around from 1,000 to 1,200 children diagnosed with HIV, all of them infected through MTCT. Vertically HIV-infected children have unique significance because the HIV infection takes place in immature immune and nervous systems, which is of particular interest to researchers [10-12].

A treatment immediately used after the birth has made a tremendous difference to the HIV-infected children. At the present moment it is not known how the HAART era will affect the health and the survival of these children [13]. Today HIV-infected infants and children survive to adolescence and adulthood. The challenge of providing HIV care involves both acute and chronic, lifelong care. Families and health providers are interested in all aspects of child’s progress and how HIV may affect her/his performance in everyday tasks. In Spain the first HIV-infected child was diagnosed in 1980. At present he is 32 years old [14].

There is no the National Registry of HIV-infected children and adults in Spain. The only Registry, which exists, is the National Registry of AIDS. The main objective of the Cohort of the Spanish Paediatric HIV Network (CoRISpe) is to contribute to furthering scientific knowledge on paediatric HIV infection by providing the demographic (the country of birth, nationality of the parents, the date of birth, the date of diagnosis, the day of death, sex, race, etc), sociopsychological (educational level of parents, foster parents, social status of the childs family, etc), clinical (HIV-progression, different illness related to HIV infection, the percentage and absolute number of CD4 and CD8, the level of plasma viral load, etc) and laboratory data (the level of cholesterol, triglycerides, transaminases, leucocytes, limphocytes, etc). Its aim is to enable high-quality research studies on HIV-infected children.

### The role of the CoRISpe

**Figure 1:**
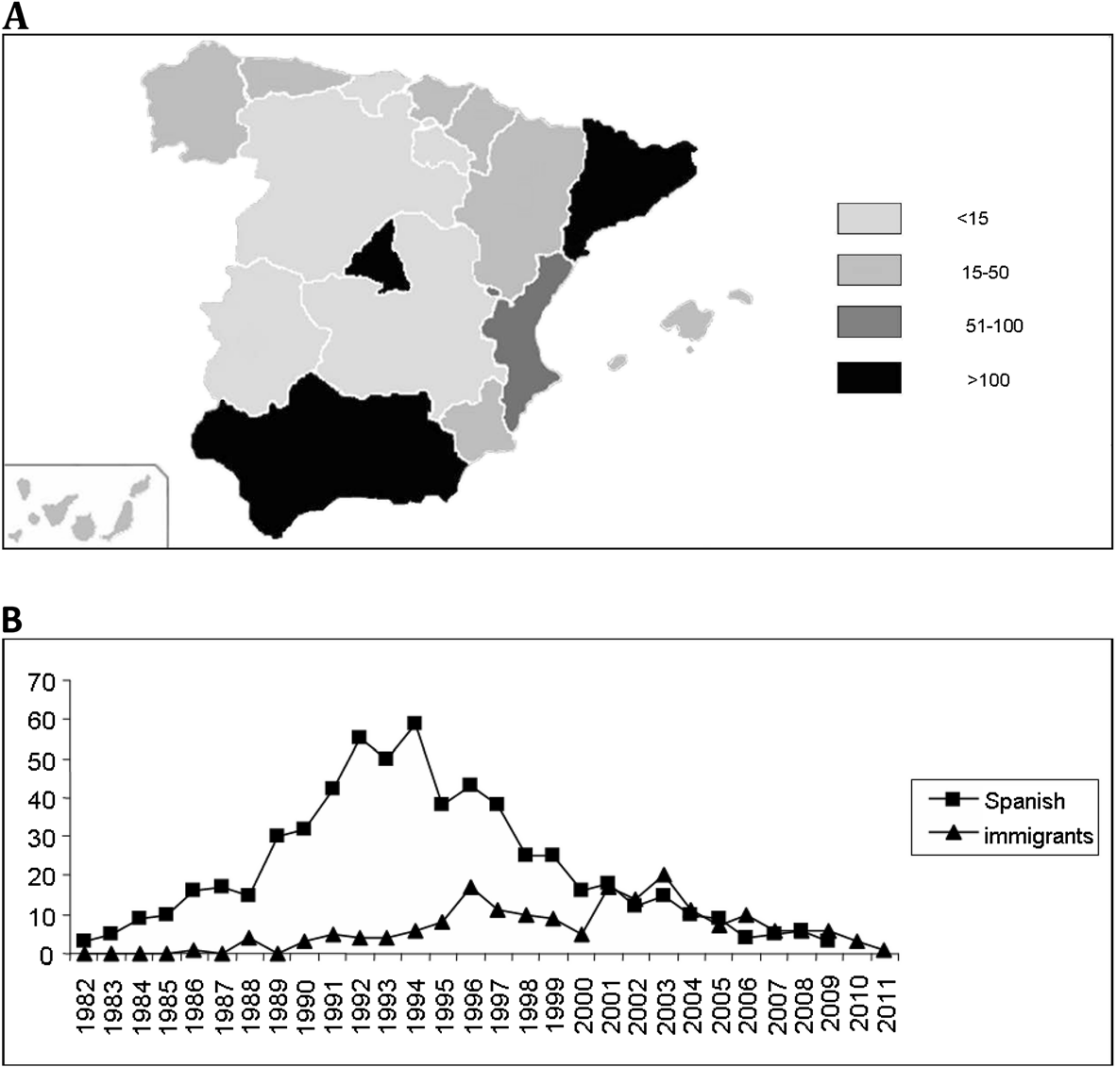
**A. The number of HIV-infected children for each Autonomous Community is represented in the colours indicated.** The highest number of HIV-infected children can be seen in Catalonia, Madrid Community and Andalusia. **B**. Spanish: HIV-infected children born in Spain parents: Immigrats: immigrants or the HIV-infected children of the immigrants.

The CoRISpe was founded in 2008 and was set up in accordance with the Spanish official law on protection of personal data [15]. We would like to emphasize the fact that it is difficult to find an electronic database (CoRISpe) containing demographic, sociopsychological, clinical and laboratory data of HIV-infected children nationally, mainly because these children are widely spread in various cities and are taken care of by different paediatricians. At the moment there are 84 hospitals with HIV-infected children in Spain and 75 of these hospitals are included in the CoRISpe representing geographically almost the entire territory of Spain (the highest number of HIV-infected children can be seen in Catalonia, Madrid Community and Andalusia; Figure 1A). These hospitals are responsible for entering the dataset into the electronic database. Retrospective data have been collected since 1995 and prospective data since 2008. There are only 7 hospitals, which treat HIV-1 infected children not included in the CoRISpe.

To obtain a database of high quality we contact all paediatricians in Spain, and explain the objectives of the CoRISpe project trying to encourage co-operation. The CoRISpe electronic database allows remote data entry by clinicians themselves and is password protected. The confidentiality and security of the patients’ data are strictly guarded and respected [15].

### The structure

The CoRISpe consists of the Steering Committee, Ethical Committee and two coordinators in the position of directors whose responsibility is to guarantee the correct performance of the CoRISpe. The Steering Committee represented by clinical researchers and participating institutions has created the basic regulations for the Internal Organization of the CoRISpe and participates in the scientific reviews of the procedures.

The Ethical Committee revises the agreements on the patients’ consents and/or assents made with different hospitals. All research projects presented in the CoRISpe have to be approved by the Ethical Committee.

The CoRISpe also has a data manager responsible to the Coordinators who clarifies any queries and gives professional advice. To increase the quality of the dataset, regular trips on a three-month basis are made by the data manager to the hospitals participating in the CoRISpe.

### The way the CoRISpe works

To provide data to the CoRISpe the parents or a legal representative of an HIV-infected child must sign an informed consent form in which the discomforts associated with obtaining the clinical and laboratory data as well as the research objectives are clearly explained. The CoRISpe respects the Declaration of Helsinki, which states that although parents or a legal representative of a child sign the consent, a child is required to give her/his assent for the participation in the study if s/he is able to do so. If after having read the assent, a child decides not to participate in the study, this decision must be respected. These informed consent and assent forms must be approved by the Ethical Committee. As a general rule, the consent can be cancelled at any time [16,17].

All the participating hospitals send to the CoRISpe the retrospective (since 1995) and prospective (since 2008) dataset of the HIV-infected children, who fulfill the inclusion criteria and have been in the clinical follow-up through the online application. By the inclusion criteria we understand: 1.The age -- younger than 18 years old at the moment of the diagnosis in the Paediatric Unit; 2. The Diagnosis of the HIV infection confirmed; 3. The predicted follow-up in the hospital and 4. The children who have seen a paediatrician at least once since 1st of January 1995. The follow-up visits of the children take place every three months and are fixed by paediatricians. There are records for teenagers and adults in the database [18]. The follow-up of each child ends when a child dies or changes to a no-CoRISpe hospital.

The electronic database applications have been designed in accordance with the norms of data protection in force and guarantee the security access pursuant to Ley Orgánica 15/1999, de 13 of December [15]. Each paediatrician has an exclusive user account, which enables the user to access her/his own data and to update the data.

The CoRISpe, which is located in the Hospital General Universitario Gregorio Marañon, provides a profound quality internal control on a regular basis. The potential errors of CoRISpe data are returned to each participating hospital for checking every 6 months. A specialised agency carries out a CoRISpe external audition verifying if the database information is in accordance with clinical records of the HIV-infected patients. This audition is performed annually on 10% of the clinical records in each hospital.

Any researcher who is a member of the RED RIS [19] or anyone who is in collaboration with a member can apply for the CoRISpe dataset as long as the project is scientifically, technically and ethically viable. A researcher must complete a data release application to receive data. This application must be submitted to the members of the Steering Committee for an evaluation. If the project is approved of, a researcher signs a release agreement with the coordinators of the CoRISpe where the commitments of both parts regarding the usage of information, criteria of follow-ups, authorship and spread of results are stated. The CoRISpe is responsible for supplying the dataset needed to carry out projects. Once the above data have been given, the principal researcher sends a scientific report containing the project results once a year so that the CoRISpe could keep updated records on all the projects. The CoRISpe does not charge any fee for providing data to research groups.

There is a special authorship policy for publications as well as for abstracts/ communication presented to the congress and approved by the Steering Committee. Regardless the amount of work each writer puts, s/he will appear on the list of authors of a given article in proportional turns among all the researchers of the participating centres.

The CoRISpe organises a general meeting with the Steering Committee and paediatricians from all the parts of Spain once a year to evaluate the stage of the objective and to plan new aims, projects, studies and numbers of research. Also, the CoRISpe prepares professional training, symposia, conferences and scientific fora.

### Transition from paediatric to adults units

#### The criteria to establish the age of HIV-infected children to be transferred to the Adult Units

The chronological and cognitive limits of the adolescent period are unclear as there are several theoretical and practical approaches [20]. WHO recognizes the adolescent age as the period between 12 and 21 years. The American Academy of Paediatrics between 13 and 21 years [21], whereas for the CDC adolescence lasts up to the age of 24 [22]. In Spain, adolescence ends at the age of 18 when an individual acquires legal autonomy.

The transfer of an adolescent suffering from a chronic disease (heart disease, cystic fibrosis, diabetes) to the Adult Unit is usually difficult [23-28]. Therefore, the records stored by the transfer units of those adolescents suffered from chronic diseases can be used as a point of reference for the transfer units of HIV-infected children taking into consideration the scarcity of data of the last ones [20,21].

#### The way to obtain the dataset from the Adult Units

It is very important to know what happens with the vertically HIV-infected children who have been transferred to the Adult Units. These vertically HIV-infected adults have different social, psychological, clinical, immunological, neurological and virological characteristics from the patients infected by different ways [29-32]. To obtain data from the Adult Units a paediatrician contacts an adult clinician to explain the objective of the CoRISpe and then their collaboration begins. A coordinator of the CoRISpe contacts an adult clinician and requests him to complete the online form.

#### Building efficient contacts between paediatricians, adult clinicians and patients

In the first year the clinical contact between a paediatrician and an adult clinician and a child who is in the process of becoming an adult patient must be well established to work efficiently. Very often the second year brings confusion because a HIV-vertically infected adult, not a child any more, is entitled to choose her/his doctor and to change a hospital. At that moment the contact between a paediatrician and a child is broken. In some cases it is difficult to find an adult patient and match her/his data with the data when s/he was a child. Therefore, at present we have a low level of follow-up of vertically infected young people once they are transferred to the Adult Units.

### General data

The entire population of HIV-1 infected children calculates for 1,100 to 1,200 in Spain. Up to now the CoRISpe has recruited 838 patients. Some of the patients of 262-362, which is a difference between 1,100-1,200 and 838 has not obtained the Ethical Committee approval and some of them has not signed the inform consent because they do not want to participate in the CoRISpe. Out of 838 patients 802 (95.7%) were vertically HIV-infected. At this moment we cannot calculate the rate of loss-to-follow-up of the coRISpe because we do not have the number of dead patients and those who interrupted the treatment. We are in the process of getting the data from paediatricians. The data of the 802 vertically HIV-infected patients included in the CoRISpe are shown in the Table 1. Vertically HIV-infected patients according to the year of birth and if the patients were born in Spain or somewhere else (Figure 1B).

**Table 1:** Demographic, immunological and virological profile of the vertically-HIV infected patients in the CoRISpe

	**Group A <18 years**	**Group B ≥18 years**	**Total**
	**N=520**	**N=282**	**N=802**
Age (years) mean (range)	12,6 (0,04-17,98)	22 (18,1-29,7)	15,1 (0,04-29,7)
Sex: N (%)			
Male	241 (46,3)	127 (45)	368 (45,9)
Female	279 (53,7)	155 (55)	434 (54,1)
Origin: N (%)			
Spain	353 (67,9)	261 (92,5)	614 (76,6)
Sub-Saharan Africa	102 (19,6)	7 (2,5)	109 (13,6)
Latin America	39 (7,5)	7 (2,5)	46 (5,7)
North Africa	10 (1,9)	3 (1,1)	13 (1,6)
East Europe	9 (1,7)	0 (0,0)	9 (1,1)
Asia	4 (0,8)	1 (0,3)	5 (0,6)
West Europe	3 (0,6)	3 (1,1)	6 (08)
Time of identification of infection			
At Birth	79 (15,2)	11 (3,9)	90 (11,2)
After Birth (asymptomatic)	264 (50,8)	160 (56,7)	424 (52,9)
After Birth (symptomatic)	151 (29)	87 (30,9)	238 (29,7)
Unknown	26 (5)	24 (8,5)	50 (6,2)
Group age at 1^st^ presentation (years)			
<1	316 (60,8)	104 (36,9)	420 (52,3)
1-5	150 (28,8)	132 (46,8)	282 (35,2)
≥6	54 (10,4)	46 (16,3)	100 (12,5)
Group year of 1^st^ presentation (years)			
1975-1990	0 (0,0)	73 (25,9)	73 (9,1)
1991-2000	289 (55,6)	199 (70,6)	488 (60,8)
2001-2011	231 (44,4)	10 (3,5)	241 (30,1)
Group age at most recent follow-up visit (years)			
0-5	70 (13,5)	0 (0,0)	70 (8,7)
6-11	209 (40,2)	0 (0,0)	209 (26,1)
12-17	241 (46,3)	0 (0,0)	241 (30)
≥18	0 (0,0)	282 (100)	282 (35,2)
CDC: N (%)			
N-A	270 (51,9)	91 (32,3)	361 (45)
B	127 (24,4)	106 (37,6)	233 (29,1)
C	123 (23,7)	85 (30,1)	208 (25,9)
Co-infections: N (%)			
HCV	24 (4,6)	31 (11)	55 (6,9)
HBV	7 (1,3)	3 (1,1)	10 (1,3)
CMV	19 (3,7)	3 (1,1)	22 (2,7)
Other	4 (0,8)	2 (0,7)	6 (0,7)
No Co-infections	466 (89,6)	243 (86,1)	709 (88,4)
Immunological status (%CD4) N (%)			
<15%	9 (1,8)	20 (7,1)	29 (3,6)
15-25%	49 (9,4)	49 (17,4)	98 (12,3)
>25%	462 (88,8)	183 (64,9)	645 (80,4)
Unknown	0 (0,0)	30 (10,6)	30 (3,7)
Viral load (copies/ml) N (%)			
<50	341 (65,6)	134 (47,5)	475 (59,2)
50-10000	92 (17,7)	83 (29,4)	175 (21,8)
>10000	27 (5,2)	36 (12,8)	63 (7,9)
Unknown	60 (11,5)	29 (10,3)	89 (11,1)

We divided the 838 HIV-infected children into two groups. The group A: 536 children <18 years old that had a follow-up in the Paediatric Units. Out of 536 patients 520 (97%) were vertically HIV-infected of average 12.6 years old (range from 0.04 to 17.98 years old). 53.7% out of 520 were girls. 353 (67.9%) out of 520 patients were born in Spain from Spanish parents, 167 (32.1%) out of 520 were immigrants or the children of the immigrants (Table 1). Fifty-four out of 520 (10.4%) children were also co-infected by other viruses such us HCV, HBV or CMV.

The group B: 302 HIV-infected patients ≥18 years of age were transferred to the Adult Units. Out of 302 HIV-infected patients 282 (93.4%) were vertically HIV-infected of average 22 years old (range from 18.1 to 29.7 years old). 155 (55%) out of 282 were girls. The average age of the patients followed up in the Adult Units was 2.38 years (range from 1.5 to 4.8 years old). 261 (92.5%) were born in Spain from Spanish parents and only 7.5% were immigrants or the children of the immigrants (Table 1).

At the moment we are in the process of getting data from the Adult Units as so far we have only been working with the Pediatric Units. The antiretroviral treatment of only 484 children out of 520 has been available. In 21 HIV-infected children the treatment was interrupted and 3 did not receive any antiretroviral treatment. 460 patients out of 484 received the HAART. 225 (48.9%) patients received 2 nucleoside analogs and protease inhibitors. Moreover, 141 (30.7%) patients received 2 nucleoside analogs and 1 non analog, 42 (9.1%) patients 1 nucleoside analog, 1 nucleoside non analog and 1 protease inhibitor, 14 (3%) patients took 3 nucleoside analogs, 24 (5.2%) patients received various treatments including CCR5 enter inhibitor or integrase inhibitors. Finally, 14 (3%) patients took monotherapy with IPs. The most used antiretroviral drugs in the CoRISpe were Lopinavir/r (51.1%), 3TC (40%), ABV (39.1%) and FTC (30.2%) [13].

It is important to point out that there is a close co-operation between the CoRISpe and the Spanish Paediatric HIV BioBank [16]. We have done seven research projects with the data provided by the CoRISpe and/or with the samples supplied by the BioBank. The results of all these projects are available to the paediatricians participating in the CoRISpe with a view to improving the state of health of their patients [8,33-44].

## Conclusions

To have the CoRISpe electronic database available has been a very important issue in the research of vertically HIV-infected children since the first years of the pandemic. We have a follow-up of the HIV-infected children in the CoRISpe database of the Paediatric Units and we have been working with these data obtaining interesting results as it is seen in a great number of our published articles [8,16,33-44]. Moreover, the CoRISpe has been collaborating with different European Cohorts (EPPICC, COHERE and PENTA) for many years.

The CoRISpe represents a novel approach to HIV research that might be of general interest not only for basic and clinical research teams working with HIV, but also for those groups trying to establish large networks focused on researching specific clinical problems. It also represents a model to stimulate cooperative research on specific clinical issues.

The main objective of this article has been to present the notion of the CoRISpe, its role and to describe how it works. The new exciting era of personalised medicine is accompanied by the rise in the importance of the databases and the biobanks. The quality of the data of the CoRISpe, the number and quality of the samples stored in the Spanish Paediatric HIV BioBank [16] are required to fulfill the needs of personalised medicine. In the coming years the databases and biobanks will represent the most important structures through which a high number of researches in the majority of scientific fields will be carried out.

## Abbreviations

CoRISpe: Cohort of the Spanish Paediatric HIV Network; MTCT: Mother-to-child transmission; HAART: Highly active antiretroviral therapy.

## Competing interests

We declare that we have no conflict of interest.

## Authors’ contributions

MªIJ and MAMF conceived the study, designed and wrote the manuscript. MªIJ, MAMF and SJ designed the CoRISpe database. SJ and ME provided and analyzed the patients`s data. MªIJ, MAMF, CF, SJ, MN and PS prepared the figures and tables. MªIJ, MAMF, CF, MN and PS discussed the final manuscript. All authors read and approved the final manuscript.

## Pre-publication history

The pre-publication history for this paper can be accessed here:

http://www.biomedcentral.com/1471-2334/13/2/prepub
